# Optical Mapping of Pacing‐Elicited Slow Waves in the Swine Stomach: Role of Virtual Electrodes

**DOI:** 10.1111/nmo.70340

**Published:** 2026-05-05

**Authors:** Hanyu Zhang, Haley N. Patton, Nipuni D. Nagahawatte, Bijay Guragain, Leo K. Cheng, Gregory P. Walcott, Jack M. Rogers

**Affiliations:** ^1^ Department of Biomedical Engineering, School of Medicine and School of Engineering University of Alabama at Birmingham Birmingham Alabama USA; ^2^ Auckland Bioengineering Institute University of Auckland Auckland New Zealand; ^3^ Department of Medicine/Cardiovascular Diseases University of Alabama at Birmingham Birmingham Alabama USA

**Keywords:** electrical stimulation, gastric, polarization, virtual electrode, voltage sensitive dye

## Abstract

**Background:**

Stomach contractions are coordinated in part by bioelectric slow waves (SW). Dysfunctional SWs are associated with motility disorders. Electrical pacing is a potential strategy for managing motility disorders but remains poorly understood with inconsistent efficacy.

**Methods:**

We used a newly‐developed optical mapping method to image gastric pacing in 4 pigs (35.0 ± 1.3 kg). The method imaged transmembrane potential, primarily from the circular smooth muscle layer, with high spatiotemporal resolution. We delivered unipolar pacing pulses to the serosal surface or to the luminal side of the circular muscle layer. Pulses were 100 ms in duration with 4 or 8 mA amplitude.

**Key Results:**

Pacing elicited transmembrane potential polarization patterns consistent with bidomain theory: For cathodal pacing, there was an elongated depolarized region (virtual cathode) oriented orthogonally to the smooth muscle fibers and centered on the electrode. It was flanked on either side by hyperpolarized virtual anodes. For anodal pacing, virtual electrode polarity was reversed. Of 175 pulses, 18% induced SWs that activated the entire mapping region. The remaining pulses failed completely (38%) or induced SWs that only partially activated the mapping region (43%). All SWs initiated from virtual cathode sites approximately 1 cm from the pacing electrode and not from the electrode site itself.

**Conclusions and Inferences:**

These results suggest that close to the electrode, pacing pulses inhibited the network of interstitial cells of Cajal (ICC) that propagates SW. SWs may have initiated when the ICC network was activated by depolarized smooth muscle in the virtual cathodes remote from the electrode.

## Key Points

- Membrane polarization patterns caused by electrical pacing pulses applied to the stomach were imaged for the first time.
- The tissue's response to pacing was complex, and regardless of pacing polarity, included adjacent regions of depolarized and hyperpolarized tissue known as virtual electrodes.
- Electrical slow waves elicited by pacing started from depolarized virtual cathodes approximately 1 cm from the pacing electrode site and not from the electrode site itself.

## Plain Language Summary

Stomach contractions are triggered by electrical waves that move through the stomach wall. Problems with these waves lead to stomach motility disorders. Electrical pacing is a potential way to improve the stomach's electrical function—just like in the heart—but it does not always work well. We used a new imaging method to see how electrical pulses affect the stomach. We found that pulses create complex patterns where small regions of “depolarized” muscle (more likely to start an electrical wave) are next to regions of “hyperpolarized” muscle (less likely to start a wave). These regions surround the electrode that the pulse came from. When new waves start, they come from the depolarized regions that are about 1 cm from the electrode and not from the electrode itself. These results may explain why the stomach is hard to pace and gives hints into new strategies to improve the stomach's electrical function.

## Graphical abstract

Unipolar electrical pacing stimuli delivered to the stomach wall induce adjacent regions of both depolarized and hyperpolarized tissue known as virtual electrodes. Electrical slow waves elicited by pacing start from depolarized virtual electrodes approximately 1 cm from the pacing electrode site and not from the electrode site itself.

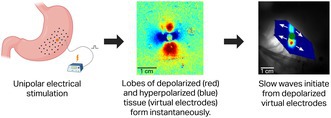

## 1 Introduction

Peristaltic contractions in stomach are regulated in part by rhythmic bioelectric slow waves (SW) [1]. Dysrhythmic SW activity is associated with multiple gastric motility disorders with sometimes debilitating symptoms such as vomiting, nausea, and bloating [2]. Gastric motility disorders are prevalent in modern communities. It is estimated that gastrointestinal functional motility disorders affect over 10% of the global population [3, 4], posing significant burdens to communities and families. Gastric pacing seeks to restore rhythmic SW activity and is a promising method for gastric motility disorder management [5]. However, gastric pacing shows inconsistent efficacy and the mechanism of action remains poorly understood [5, 6].

Electrical mapping techniques using high‐resolution extracellular electrode arrays have been used to study the response of the tissue to pacing [7, 8, 9] However, electrical pacing artifacts obscure the immediate response of the tissue to the pacing pulse, making it difficult to image direct pacing effects [10]. We recently implemented and validated an alternative technology, optical mapping, for gastric preparations [11, 12]. Optical mapping uses voltage sensitive dyes (VSDs) to convert membrane potential (Vm) into fluorescence signals, which can be recorded using high speed cameras. Because optical mapping is immune to recording artifacts from electrical stimulation, directly images Vm rather than extracellular potentials, and typically has higher spatial resolution than electrical mapping, it is well‐suited to imaging electrical stimulation effects. It is widely used to study cardiac pacing and defibrillation [13].

Electrical stimulation in cardiac tissue is often analyzed in terms of bidomain theory, which holds that myocardium is composed of two interpenetrating domains: the intracellular space and the extracellular space. Both domains are anisotropic, meaning that electrical conductivity is different along cells than across cells. When a stimulus is delivered through a unipolar electrode in the extracellular space (with a distant reference electrode), the electrical potential in the extracellular space falls off spatially in an elliptical pattern. Directly under the electrode, capacitive current enters the intracellular space and, like in the extracellular space, intracellular electrical potential falls off in an elliptical pattern. In myocardium, the anisotropy *ratio* (conductivity along vs. across fibers) is different in the two domains; therefore, the elliptical potential decay patterns in the intra‐ and extracellular spaces have different shapes. These differing potential patterns cause the transmembrane potential—the difference between intra‐ and extracellular potential—to have complex spatial patterns. Typically, for a cathodal stimulus (negative charge flowing from the electrode into the extracellular space), there is an elongated “dogbone” shaped region of depolarization under the electrode that is aligned with the muscle fiber orientation. Flanking the depolarized region orthogonal to the fibers are two hyperpolarized regions. These regions are called virtual electrodes. With anodal stimulation, the polarity of the virtual electrodes is reversed. These virtual electrode patterns were first predicted in the late 1980s using numerical simulations that treated cardiac tissue as distinct, continuous, interpenetrating intra‐ and extracellular domains. They were subsequently confirmed using optical mapping [14].

In our recent preliminary study, we used in vivo gastric optical mapping to show that unipolar point pacing pulses on the serosal surface produced virtual electrodes consistent with bidomain theory just as electrical stimulation does in the heart [15]. In the present study, we further show that the virtual electrode patterns are aligned with the fiber orientation in the circular smooth muscle layer. In addition, we relate pacing‐elicited SWs with the virtual electrodes. We identify different modes of pacing success and failure, which together suggest that SW formation following electrical pacing is governed by multiple mechanisms.

## 2 Methods and Materials

### 2.1 Animal Preparation

All animal protocols were approved by the University of Alabama at Birmingham Institutional Animal Care and Use Committee (APN21867), and all animal protocols were in accordance with the Guide for the Care and Use of Laboratory Animals.

**FIGURE 1:**
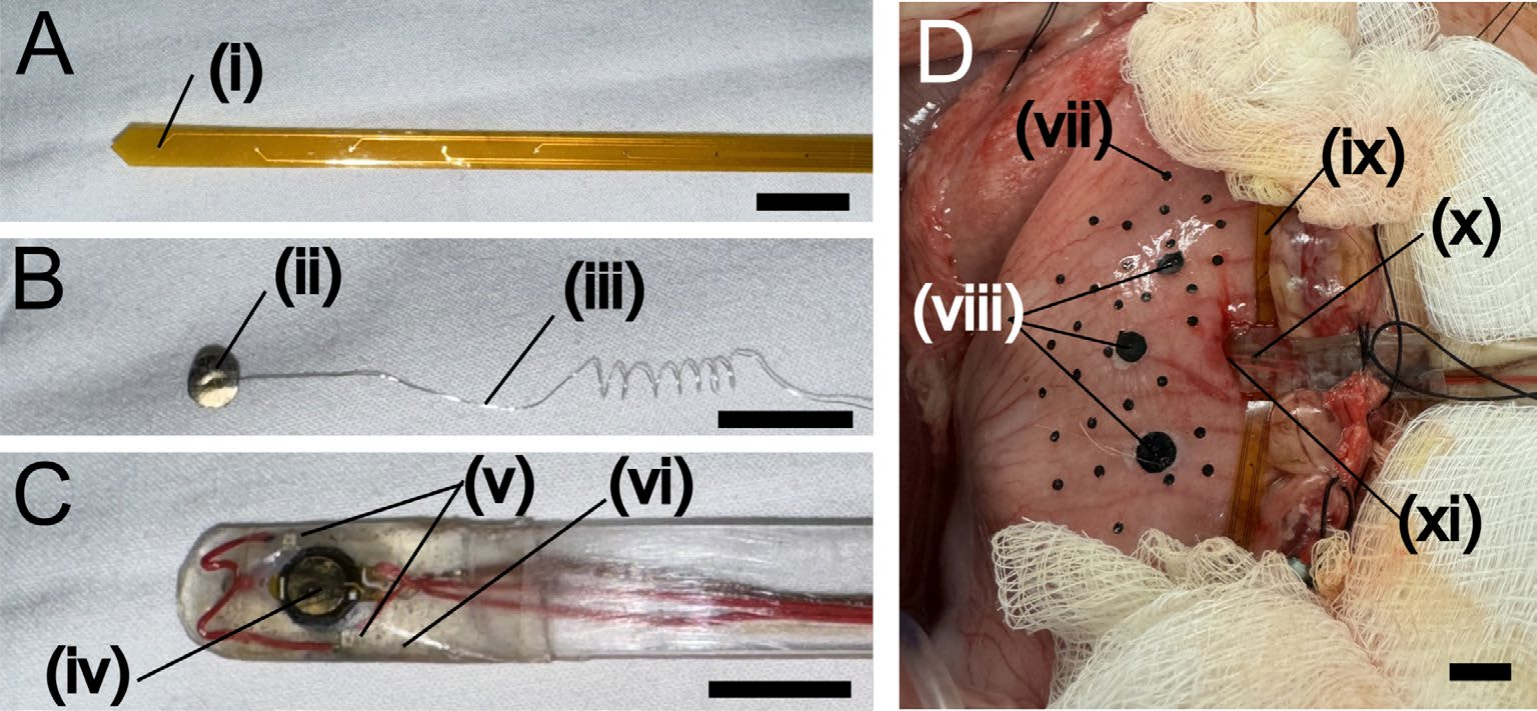
Experimental setup. (A) 1 × 8‐channel flexible printed circuit electrode array for real‐time slow wave monitoring. (i) A recording electrode (0.5 mm diameter). (B) Serosal pacing electrode. (ii) Gold‐plated pacing electrode (4 mm diameter). (iii) Insulated silver wire (0.1 mm diameter, California Fine Wire Co.). (C) Submuscular pacing electrode. (iv) Gold plated pacing electrode (4 mm diameter). (v) Red LEDs (SML‐212U2TT86A, Rohm Semiconductor). (vi) Clear epoxy for component embedding and insulation. (D) In vivo stomach preparation. (vii) Fiducial markers for motion tracking. (viii) Pacing site markers. (ix) 1 × 8‐channel flexible printed circuit electrode array placed along the greater curvature. (x) Submuscular pacing electrode inserted into a submuscular pocket through an incision (xi). Scale bars represent 1 cm.

Four healthy domestic farm pigs (1 male and 3 female), weighing 35.0 ± 1.3 kg were used in this study. Surgical preparation was similar to our previous publication [12]. Briefly, animals were fasted overnight prior to each experiment. Anesthesia was induced with intramuscular injection of atropine/telazol/xylazine (0.4/4.4/4.4 mg/kg) and maintained with inhalation isoflurane (2.5%–5% in 100% oxygen). The abdominal cavity was opened with a midline laparotomy and held open with a ring retractor (Alexis O, Applied Medical). The right gastroepiploic artery (RGE) was cannulated for VSD infusion with a 21‐gauge catheter and tied off. Blood perfusion to the stomach was maintained by the left gastroepiploic artery (LGE), which anastomoses with the RGE. Twenty to thirty fiducial markers (2 mm diameter, ~10 mm spacing) were attached to the anterior serosal surface with tissue glue (Vetbond, 3 M). The region spanned by markers (termed the mapping region) was roughly 4 × 7 cm and located in the mid‐corpus to proximal antrum. A 1 × 8 linear array of electrodes printed on a flexible substrate (Figure 1A) was placed on the anterior serosal surface along the greater curvature. The electrode array was connected to an electrical mapping system (LabSystem Pro, Bard Electrophysiology). Signals were bandpass filtered (0.5–10 Hz) and displayed on a monitor. They were used to identify the presence, frequency, and direction of SWs in real‐time during the experiment, but were not used for further analysis. When the stomach was not being manipulated or imaged, the incision was covered with plastic wrap, a blanket, and warming pads to maintain warmth and moisture. Animals were euthanized with KCl while under anesthesia on completion of the study.

### 2.2 Optical Mapping

In vivo optical mapping was carried out similarly to our previous publications [11, 15]. Briefly, we used the VSD Di‐5‐ANEQ(F)PTEA (ElectroFluor630P, Potentiometric Probes), which was dissolved in 100% ethanol (1 mg/mL) and stored at −20°C. On the day of the experiment, stock solution was diluted into 15 mL oxygenated Tyrode's solution (T2145, Sigma‐Aldrich) for final VSD concentration of 50 μmol/L. The dye bolus was slowly infused into the RGE catheter over 1 min. During the infusion, the LGE was temporarily clamped to provide back pressure and direct the dye solution to the area of interest. After the dye infusion, the clamp remained on the LGE for 1–2 min to allow dye incubation. To image Vm, fluorescence was elicited by alternating green and amber LED light that was switched with camera frames for time‐interlaced excitation ratiometry [16]. Fluorescence emission was recorded by a high‐speed camera (MiCAM03, SciMedia) fitted with either a 35 mm F/1.4 lens (Rokinon) or a 50 mm F/1.4 lens (Samyang) at 300 Hz with 256 × 256 spatial resolution.

### 2.3 Gastric Pacing

We performed unipolar pacing from 3 to 4 serosal sites in each animal using a custom‐made electrode (Figure 1B) that was adhered to the serosa by surface tension. To determine if virtual electrodes extended across the thickness of the muscular wall, in two animals, a small incision was made in the stomach wall and a pocket was formed between the mucosa and the smooth muscle opposite one of the serosal pacing sites. We inserted a custom‐made submuscular pacing electrode (Figure 1C) into this pocket (Figure 1D). For all pacing, the return electrode was a surface ECG electrode (Red Dot, 3 M Medical) adhered to the lateral abdominal wall.

Pacing pulses were monophasic with fixed width of 100 ms and amplitude of 4 or 8 mA, based on a previous study [17]. An isolated current source stimulator (A385, World Precision Instruments) controlled by a function generator (Model 29, Wavetek) generated the pacing pulses. The trigger signal to the stimulator was recorded by an analog input channel of the mapping camera system to register pulse timing with the optical mapping data.

### 2.4 Imaging Protocols

Ten episodes were optically mapped at each pacing site. Each episode was 1 min in duration followed by a short delay (< 1 min) to reconfigure the instrumentation for the next episode. The 1st and 10th episodes were pacing‐free baseline runs to image spontaneous SW activity. The pacing trains for the 2nd—9th episodes were configured as shown in Table S1. There were two different pacing intervals: 15 s and 5 s. The 15 s pacing interval was chosen as 20% shorter than the normal spontaneous SW interval in isoflurane‐anesthetized pig [18]. These episodes were used to image SWs elicited by the pacing pulses. The episodes with 5 s interval were used only to image the instantaneous membrane polarization patterns (virtual electrodes) generated by the pulses. Slow wave propagation was not analyzed in these episodes. Polarization patterns from all of the pulses in a 5‐s‐interval episode were averaged to improve signal‐to‐noise ratio. The 5 s interval was chosen to provide a large number of pulses within the 1‐min imaging episode while still being sufficiently long for the cell membrane to discharge after each pulse.

After completing the pacing episodes at a serosal site, a circular plastic marker 5 mm in diameter was glued to the site to document the location of the electrode (Figure 1D). After recording from all serosal sites, in two of the animals, the submuscular pocket was formed and the electrode was inserted. A pair of red LEDs on opposite sides of the electrode (Figure 1C) were visible on the serosal surface and were used to identify the position of the electrode. Once in place, the same protocol shown in Table S1 was carried out with stimulation delivered from the submuscular electrode on the luminal side of the wall and imaging acquired from the serosal side.

### 2.5 Tissue Harvest and Identification of the Smooth Muscle Fiber Orientation

After completing all optical mapping episodes, a custom‐made plastic frame (Figure S1A) was sutured to the mapping region to preserve the shape of the stomach wall during tissue harvesting and fixation. After euthanasia, 10% neutral buffered formalin (23‐253998, Fisherbrand) was quickly perfused through the RGE catheter to fix the tissue in the mapping region. The anterior stomach wall, together with the plastic frame, was then excised and immersed in 10% neutral buffered formalin for 1 week for further fixation.

After fixation, the plastic frame was removed and the fiducial and pacing site markers on the serosal surface were replaced with transmural markers by inserting Teflon tubes (30AWG, Zeus Industrial Products) through the center of each surface marker, guided by a 16G needle. Different colored Teflon tubes were used to distinguish the pacing site markers from the fiducial markers (Figure S1B). The tissue was then placed in a −80°C freezer overnight.

On the next day, the serosal layer around the pacing sites was carefully removed by abrading the frozen tissue using fine‐toothed pre‐chilled metal files (McMaster Carr) in a −20°C cold room. Because the longitudinal smooth muscular layer is very thin or almost non‐existent in our mapping region (middle corpus to proximal antrum) [19], this process exposed the circular smooth muscle layer. The tissue was allowed to thaw at room temperature and the tissue around each pacing site was stained with 0.4% trypan blue (15250‐061, Gibco) for 1 min followed by flushing with water. Trypan blue staining enhances the contrast between muscle fibers and surrounding tissue and helps identify the muscle fiber orientation. Stained tissue was imaged with a dissecting microscope (AmScope) and the orientation of the muscle fibers was noted. Each image was transformed to the mapping camera's perspective using affine transformation (rotation, translation, scale, and shear) based on the fiducial markers around the pacing site, which allowed the fiber orientation to be spatially registered with the optical mapping data.

### 2.6 Data Processing

#### 2.6.1 Visualization of SW Propagation

For the recordings with no pacing or 15 s pacing interval, optical Vm signals were reconstructed using our previously published method [11, 12]. Briefly, this method consists of two stages to remove motion artifacts from the Vm recordings: First, the motion of tissue sites was tracked using the fiducial markers. Second, excitation ratiometry was used to amplify Vm, which has opposite polarity in green‐ and amber‐elicited fluorescence, while canceling residual motion artifact, which is common to both. After removing motion artifacts, optical Vm signals were spatially filtered by a 2D moving average filter with a disk‐shaped window of radius 1–3 pixels. Then, SW deflections were normalized and color‐coded by their normalized amplitude. Similarly to our previous publication [12], slow waves induced visible contractions. However, we did not quantify the contractions in this study.

#### 2.6.2 Visualization of Virtual Electrode Polarization Patterns

The virtual electrode polarization patterns were visualized using the 5‐s‐interval pacing recordings. The onset of each pacing pulse was identified by the rising edge of the pacing trigger signal. After processing with excitation ratiometry, the 5 frames before each pulse onset (33 ms duration) were averaged and likewise for the five frames after. Motion tracking was usually unnecessary during these time intervals because any stomach contraction elicited by the pulse would not occur for at least 1–2 s after electrical activation [12]. However, motion tracking was used in a few cases in which pacing‐induced skeletal muscle contraction caused motion artifact. Subtracting the before‐onset average frame from the after‐onset average frame produced an image of the virtual electrode pattern for one pacing pulse. The virtual electrode images for all pulses in a recording (~12) were averaged to produce the final virtual electrode image for the recording.

## 3 Results

### 3.1 Virtual Electrode Polarization Patterns

**FIGURE 2:**
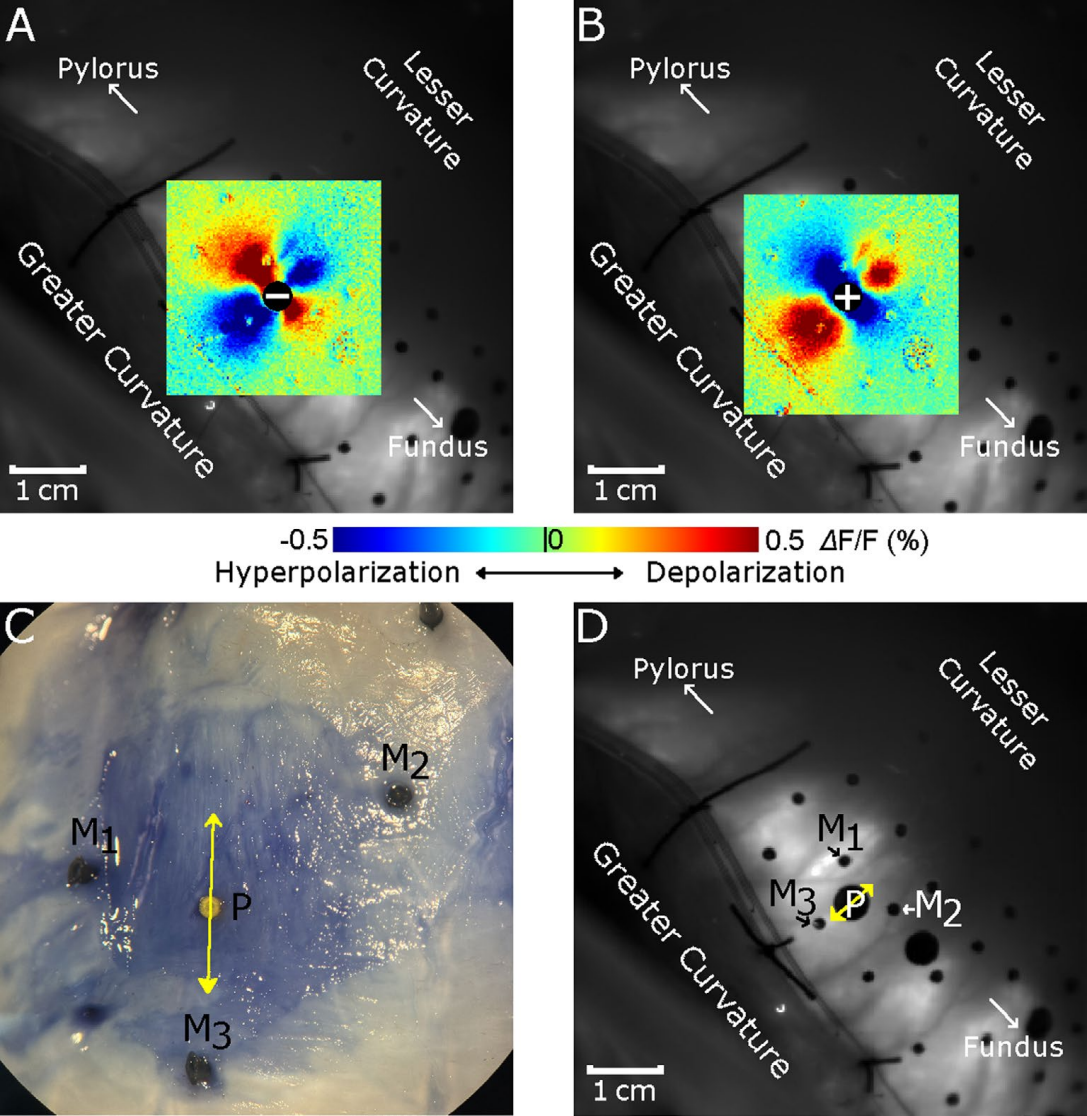
Virtual electrode patterns during serosal pacing. (A, B) Virtual electrode patterns elicited by 4 mA, 100 ms unipolar pacing stimuli. The ⊝ and ⊕ indicate the pacing location and polarity: Cathodal and anodal respectively. (C) The smooth muscle fiber orientation (yellow double arrow) of the circular layer underlying the pacing site P was identified with a dissecting microscope. The sites of the surrounding fiducial markers (M_1_—M_3_) are indicated. (D) Locations of the pacing site (P) and three surrounding fiducial markers (M_1_—M_3_) in the mapping camera view. The smooth muscle fiber orientation (yellow double arrow) was transformed from the image in C using affine transformation based on the fiducial markers.

Figure 2 shows examples of virtual electrode polarization patterns during serosal pacing from one animal. With cathodal pacing (), there was one dogbone‐shaped, depolarized central lobe (virtual cathode) with two hyperpolarized side lobes (virtual anodes) on either side of the virtual cathode's long axis (Figure 2A). Anodal pacing (⊕) produced similar virtual electrode patterns, but the lobes had opposite polarity (Figure 2B). The side lobes were aligned with the smooth muscle fiber orientation of the circular layer, which was identified in the fixed tissue (Figure 2C) and transformed to the optical mapping camera's perspective (Figure 2D).

**FIGURE 3:**
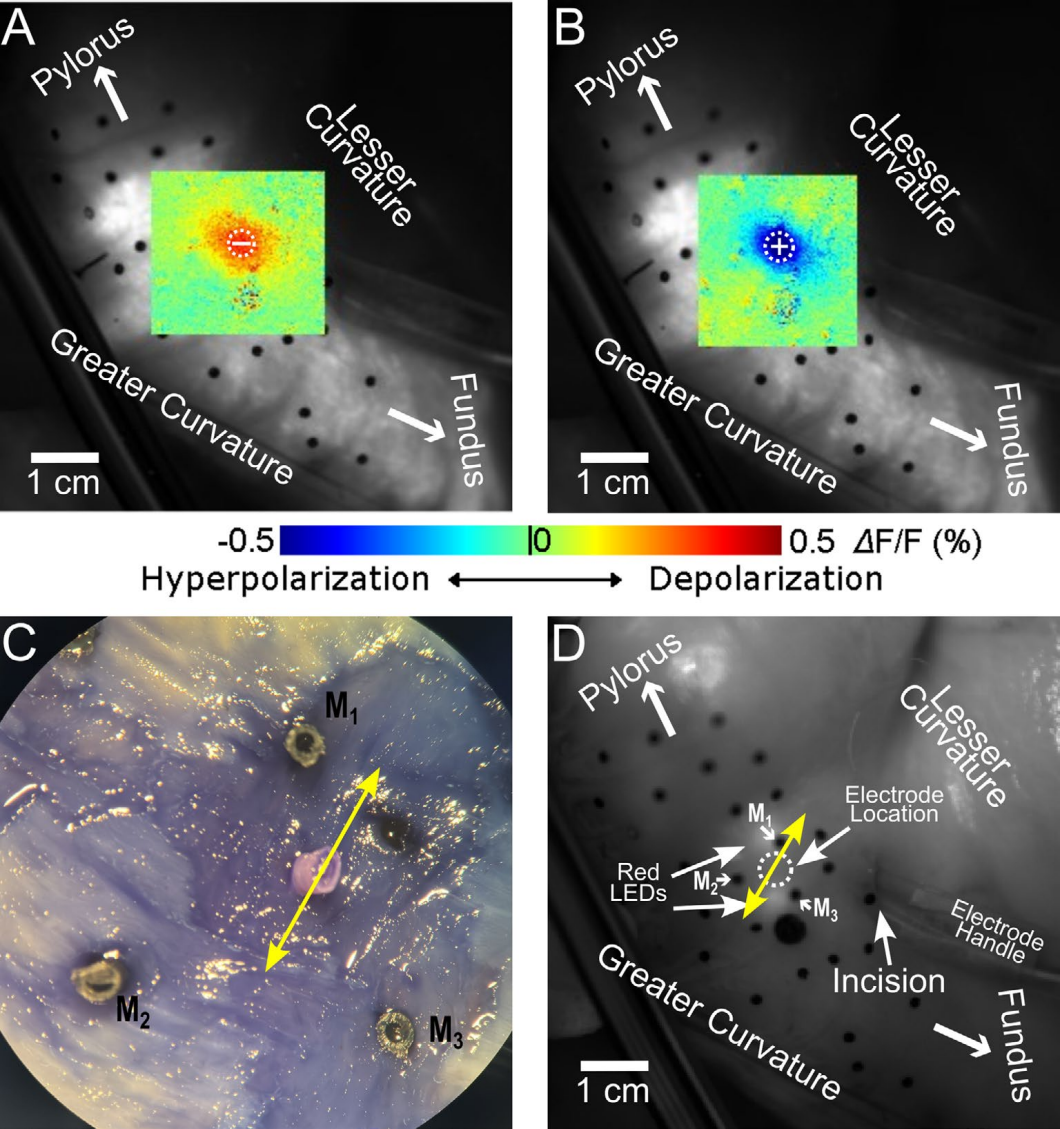
Virtual electrode patterns imaged on the serosal surface during submuscular pacing. (A, B) Virtual electrodes elicited by 4 mA, 100 ms unipolar pulses. ⊕ and ⊝ (with dashed circle) show the location and polarity of the pacing electrode on the luminal side of the stomach wall. (C) The smooth muscle fiber orientation (yellow double arrow) was identified in the fixed tissue. The sites of the surrounding fiducial markers (M_1_—M_3_) are indicated. (D) Mapping camera view showing the serosal location of the electrode (dotted circle) and surrounding fiducial markers M_1_—M_3_. The electrode location was identified by the red LEDs that were attached to either side of the electrode; the red light was visible through the muscle layers. The smooth muscle fiber orientation (yellow double arrow) was transformed from the image in C using affine transformation based on the fiducial markers.

Pacing from the electrode in the submuscular pocket produced similar polarization patterns on the serosal side of the stomach wall. However, the virtual electrodes were less distinct than during serosal pacing. Figure 3 shows examples from one animal. With cathodal pacing, there was one oval depolarized central lobe flanked by two hyperpolarized side lobes (Figure 3A). Anodal pacing produced virtual electrodes of the same shape, but with opposite polarity (Figure 3B). As with serosal pacing, the orientation of the virtual electrodes was consistent with the smooth muscle fiber orientation of the circular layer, which was identified in the fixed tissue (Figure 3C).

### 3.2 Pacing Outcomes

A total of 200 pacing pulses were delivered in the episodes that were designed to image the slow waves elicited by pacing (with 15 s pacing interval). Each 60 s episode contained 3–4 pacing pulses. Spontaneous SW activity was observed throughout the experiment in all 4 animals, which sometimes obscured the stomach's response to pacing. For this reason, 25 pulses delivered during or shortly after spontaneous SW activity were excluded from further analysis.

#### 3.2.1 Pacing Success

**FIGURE 4:**
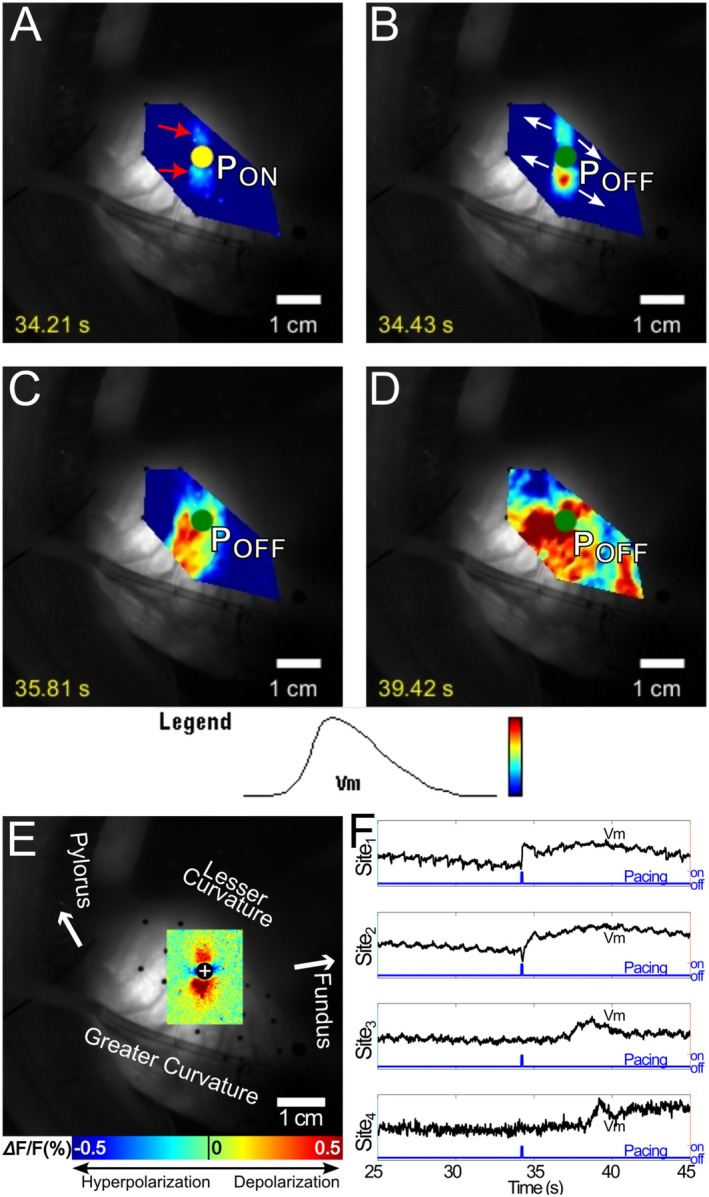
Optical mapping of type 0 success elicited by a 4 mA anodal stimulus. (A) At the onset of stimulation, depolarized virtual cathodes (red arrows) about 0.5–1 cm away from the pacing site (P) are evident. (B) The virtual cathode sites remained depolarized after the pacing stimulus, and depolarization propagated away from the two activation foci (white arrows). (C) Wavefronts from the two foci merged and propagated in both the proximal and distal directions. (D) The elicited SW propagated throughout the mapping region. A movie of this type 0 success is available as Video S1. (E) The virtual electrode pattern elicited during a 5‐s pacing interval recording with the same pacing location, polarity and strength. The virtual cathodes in E co‐located with the activation foci in A and B. (F) Optical Vm signals acquired at sites 1–4 in panel A. Site 1 was in a virtual cathode, which depolarized immediately upon stimulus delivery. The depolarization continued and formed a full‐blown SW. Site 2 was in a virtual anode; hyperpolarization during the stimulus is evident, but did not prevent later activation when the SW wavefront arrived. Sites 3 and 4 were in remote areas, where the activation was the result of SW propagation.

Of the remaining 175 pacing pulses, 32 (18%) succeeded in eliciting SWs that propagated across the entire mapping region. We observed two types of pacing success, which we termed type 0 and type 1. The distribution of successes among animals and pacing polarity is given in Table S2. In type 0 successes, the SW originated directly from the virtual electrode(s) as a result of the pacing pulse (28 of 32, 88%). Figure 4 and Video S1 show a typical type 0 success from an anodal pulse. The pulse formed two depolarized virtual cathode side lobes about 0.5–1.0 cm away from the pacing electrode that acted as activation foci (Figure 4A,B,E). SWs spread from these sites—not the pacing site—and eventually activated the entire mapping region (Figure 4C,D). Hyperpolarization of the virtual anode did not prevent its later activation when the wave reached it (Figure 4F).

**FIGURE 5:**
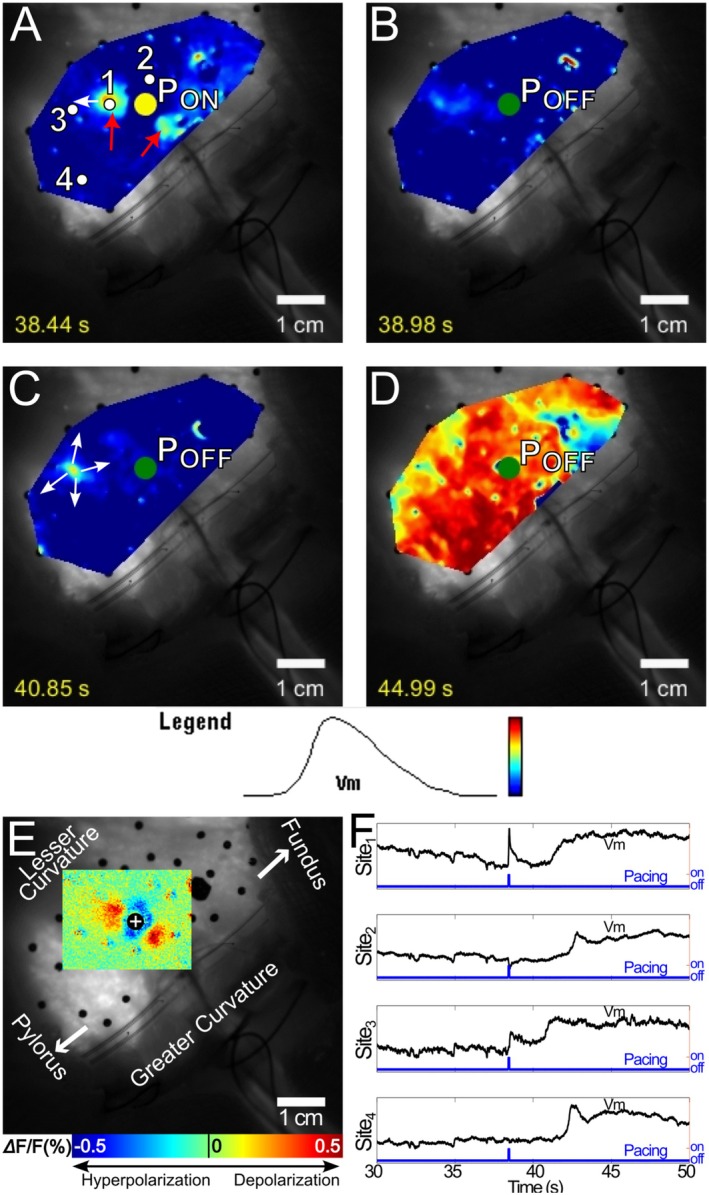
Optical mapping of type 1 success elicited by a 4 mA, anodal stimulus. (A) At the onset of stimulation, depolarized virtual cathodes (red arrows) about 0.5–1 cm away from the pacing site (P) are evident. (B) The depolarization in one virtual cathode subsided, but in the other, depolarization propagated for < 1 cm (white arrow in A) before subsiding as well. (C) Approximately 1 s later, a secondary activation focus appeared at the site at which the local activation initiated at the virtual cathode had disappeared. (D) This secondary focal activation resulted in a full‐blown SW that propagated across the entire mapping region. White arrows indicate wavefront propagation direction. A movie of this type 1 success is available as Video S2. (E) The virtual electrode pattern elicited during a 5‐s pacing interval recording with the same pacing location, polarity and strength. The virtual cathodes in E co‐located with the initial activation foci in A. (F) Optical Vm signals acquired from sites 1–4 in A. Site 1 was in a virtual cathode and depolarized immediately upon stimulus delivery, but depolarization quickly subsided. The second activation, a few seconds later, was the result of SW propagation from the secondary focus. Site 2 was in the virtual anode; hyperpolarization is visible, but did not prevent later activation when the SW wavefront arrived at this site. Site 3 was in the secondary activation focus. Both the initial and secondary activations are visible. The secondary activation occurred ~1–2 s after the repolarization of the initial activation. Site 4 was in a remote area, where the activation was the result of SW propagation.

In type 1 successes, the pacing pulse elicited a transient (~1 s or less), locally propagated (a centimeter or less), SW that quickly subsided, followed by a SW originating from a secondary focal site near the previously activated tissue. Four of 32 successes were type 1 (12%). Figure 5 and Video S2 show an example resulting from anodal pacing. A short‐lived SW initiated and propagated a short distance away from one of the depolarized virtual cathodes. The other virtual cathode failed to elicit a SW (Figure 5A,B,E). A secondary focus then arose from the terminal site of the initial short‐lived SW ~1–2 s after failure (Figure 5C). This SW eventually activated the entire mapping region (Figure 5D). Like in Figure 4, hyperpolarization of the virtual anode did not prevent later activation of that site (Figure 5F).

In both types of success, it was not necessary for activation to succeed at both virtual cathodes (side lobes during anodal pacing) or both ends of the virtual cathode (central lobe during cathodal pacing) for a SW to be initiated. Success of just one side lobe, or one end of a virtual cathode, was sufficient to start full‐blown SW activation. Figures S2 and S3 are examples of type 0 successes emerging from one end of a dogbone‐shaped virtual cathode during cathodal pacing. In Figure S3, the pacing site was on the luminal side of the smooth muscle layers.

#### 3.2.2 Pacing Failure

**FIGURE 6:**
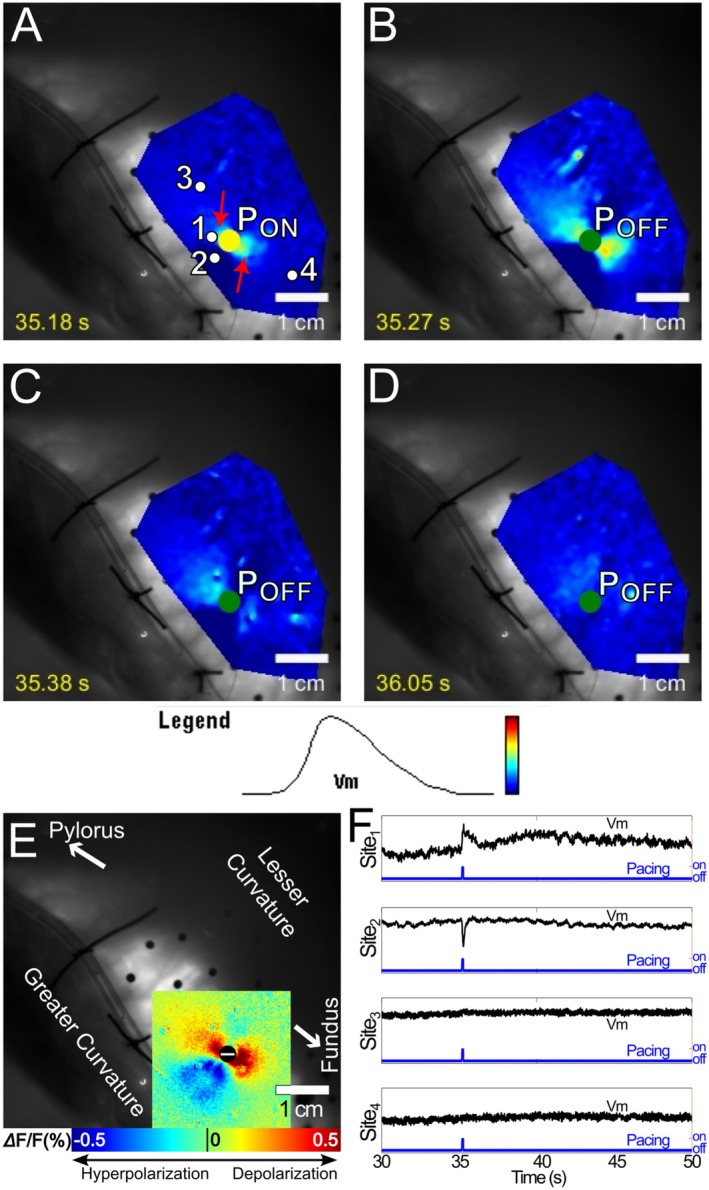
Optical mapping of type 0 failure during an 8 mA, cathodal stimulus. (A) Upon stimulation, depolarization was evident at both ends of the dogbone‐shaped virtual cathode (red arrows). (B) The depolarization reached maximum at the end of the pacing stimulus. (C, D) After the stimulus, the depolarization quickly subsided and no SW was elicited. A movie of this type 0 failure is available as Video S3. (E) The virtual electrode pattern elicited during a 5‐s pacing interval recording with the same pacing location, polarity and strength. The dogbone‐shaped virtual cathode in E co‐located with the depolarized region in A. (F) Optical Vm signals acquired from sites 1–4 in A. Sites 1 and 2 were in the virtual cathode and a virtual anode, respectively; transient depolarization and hyperpolarization were evident during the stimulus. Sites 3 and 4 were in remote areas where no polarization or transient SW was elicited.

Among the 143 failed pacing pulses, we observed three types of pacing failure, which we termed type 0, type 1, and type 2. Their distribution among animals and pacing polarity is listed in Table S2. In type 0 failures (67 of 143, 47%), the tissue polarization simply subsided. Figure 6 and Video S3 show an example of type 0 failure resulting from 8 mA cathodal pacing. A transient depolarization was evident in the dogbone‐shaped virtual cathode (Figure 6A,B,E,F), but despite the high pacing strength, depolarization quickly subsided without generating a propagating SW (Figure 6C,D,F).

**FIGURE 7:**
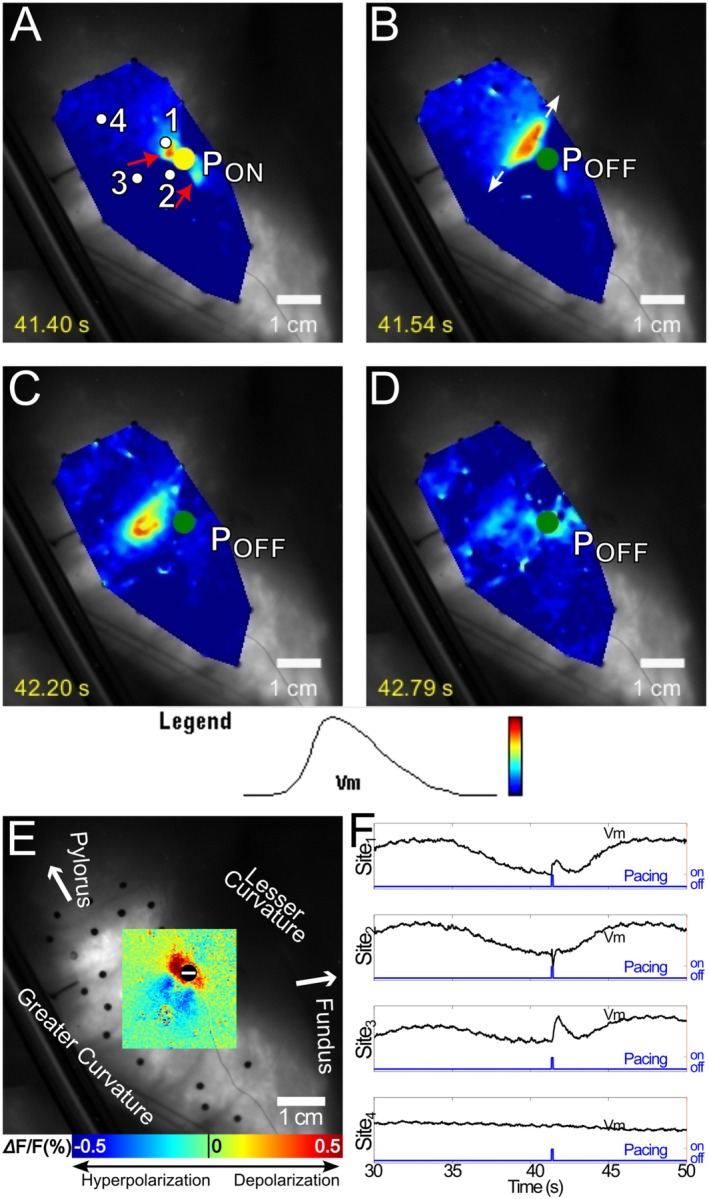
Optical mapping of type 1 failure of an 8 mA cathodal stimulus. (A) Upon stimulation, depolarization was evident at both ends of the dogbone‐shaped virtual cathode (red arrows). (B) After the stimulus, the depolarization at one end of the virtual cathode subsided. At the other end, the depolarization formed an oval‐shaped activation focus. White arrows show the wavefront propagation direction. C, D The activation propagated for a short distance (< 1 cm), then also subsided. (E) The ensemble‐averaged virtual electrode pattern elicited during a 5‐s pacing interval recording with the same pacing location, polarity and strength. The dogbone‐shaped virtual cathode in E co‐located with the depolarized region in A. (F) Optical Vm signals acquired from sites 1–4 in A. Site 1 was in the virtual cathode, which depolarized for ~1 s upon stimulation. Site 2 was in the virtual anode, which exhibited hyperpolarization. Site 3 activated after the stimulus as a result of local propagation. Site 4 was in a remote area where no stimulus polarization or SW activation was observed.

Type 1 failures (60 of 143, 42%) were similar to type 1 successes in that very short‐lived, locally propagated (< 1 cm) activations were elicited. However, these transient SWs were not followed by a focal SW as in a type 1 success. The transient SWs typically propagated anisotropically in the circular muscle fiber direction. Figure 7 and Video S4 show a typical type 1 failure following an 8 mA cathodal pulse. Depolarization at the ends of the dogbone‐shaped virtual cathode are evident (Figure 7A,E,F). Polarization at one end quickly subsided. At the other end, a transient SW was elicited and propagated in the circular muscle fiber direction for < 1 cm before also subsiding (Figure 7B–D).

Type 0 and type 1 pacing failures did not appear to be solely due to tissue refractoriness. In the cases of Figure 6/Video S3 and Figure 7/Video S4, the tissue in the mapping region had not been activated by a SW for more than 30 s before the pulse. Pacing failure could also occur just before the arrival of a spontaneous SW, during which time the tissue was ready to fire a new SW activation (Figure S4).

**FIGURE 8:**
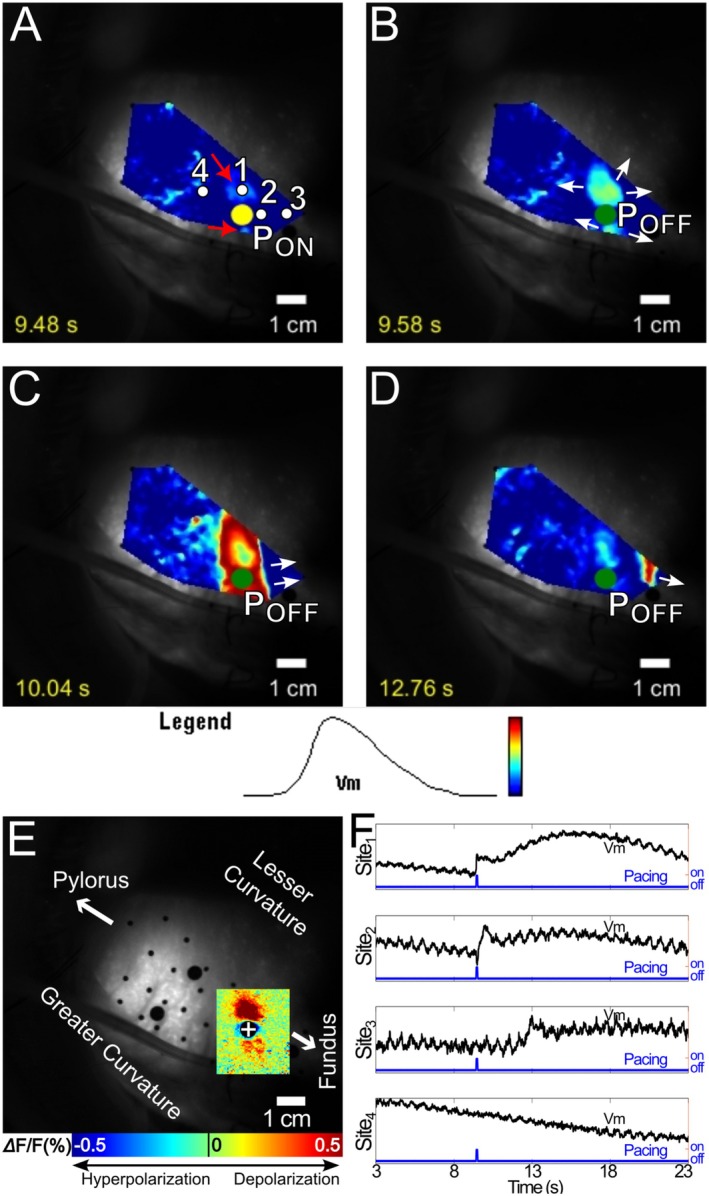
Optical mapping of type 2 failure of an 8 mA, anodal stimulus. (A) Depolarization was evident at both virtual cathodes (red arrows). (B) The depolarization of the virtual cathodes expanded into two activation foci. (C) The wavefronts from the two foci merged, but only propagated in the proximal direction. (D) SW was elicited only in the proximal half of the stomach. White arrows show the wavefront propagation direction. (E) The ensemble‐averaged virtual electrode pattern elicited during a 5‐s pacing interval recording with the same pacing location, polarity and strength. The virtual cathodes in E co‐located with the initial activation foci in A and B. (F) Optical Vm signals acquired from sites 1–4 in AR. Site 1 was in a virtual cathode. Depolarization was immediate upon stimulation and continued to form a SW. Site 2 was in a virtual anode. Hyperpolarization during the stimulus is evident, but did not affect later depolarization when the SW wavefront arrived. Sites 3 and 4 were in proximal and distal remote areas respectively. No SW was elicited distal to the stimulation site due to conduction failure.

In type 2 failures (16 of 143, 11%), regional activation on the scale of centimeters was elicited, but the SW failed to propagate to the entire mapping region. Figure 8 and Video S5 show an example of a type 2 failure. The two virtual cathodes depolarized and formed foci that merged and propagated to the right (proximally; Figure 8A–C,E,F), eventually activating all of the mapping region on that side of the pacing site. However, propagation failed in the distal direction (Figure 8D,F). Type 2 failures have also been termed “partial entrainment.” [7] They may initially resemble either type 0 or type 1 successes but are distinguished by their eventual downstream propagation failure.

## 4 Discussion

Pacing the stomach electrically is a potential therapeutic approach for gastric motility disorders [5]. However, gastric pacing has shown inconsistent efficacy in preclinical and clinical studies, which has limited its widespread clinical translation [5, 20]. Preclinical studies using high‐resolution mapping techniques have investigated mechanisms of gastric pacing [7, 8, 9]. However, when using conventional electrical mapping techniques, electrical pacing stimuli polarize mapping electrodes and saturate amplifiers, obscuring the tissue's response during and shortly after the pulse [10, 21]. Optical mapping using VSDs has been widely used in cardiac research [13] and has recently been successfully adapted for gastric mapping [11, 12]. Unique advantages of optical mapping relative to electrical mapping are its immunity to pacing artifact, ability to directly image Vm, and high spatial resolution [13]. In the present study, we were able to image the Vm response of gastric tissue from the initial stimulus‐induced tissue polarization to the eventual formation of SW.

### 4.1 Source of Optical Mapping Signals

The fluorescence signal registered at a camera pixel during optical mapping is the summation of fluorescence emitted by a small volume of tissue [22]. In our setup, the signals are estimated to originate from superficial tissue with depth on the order of a few hundred microns [12]. Our mapping region was located in the mid‐corpus to proximal antrum, where in pigs, the longitudinal smooth muscular layer is very thin—a small fraction of the thickness of the circular muscle layer [19]. A two‐dimensional network of interstitial cells of Cajal (ICC) is located in the myenteric plexus between the two muscle layers (ICC‐MY). Slow waves are generated and propagated through this network [23, 24]. Because the volume interrogated by our optical mapping system extends deeper than the thin longitudinal muscle and ICC‐MY layers, the signals acquired during optical mapping are a summation of Vm from longitudinal and circular myocytes as well as ICCs. However, we believe cells of the circular smooth muscle layer are the primary contributors to our Vm signals because of the much greater thickness of this muscle layer in this region of the stomach. In future studies, it may be possible to label different cell populations with genetically encoded calcium [25] or voltage [26] indicators, which would enable electrical function of the different networks to be separated.

### 4.2 Virtual Electrodes

The virtual electrode polarization patterns we observed had side lobes aligned with the circular muscle fiber orientation and an orthogonal central lobe. The virtual electrodes extended ~1 cm from the pacing site in the serosal plane (Figure 2). Data from submuscular pacing (Figure 3) indicate that the virtual electrodes extended across the entire muscle wall as well. These patterns are consistent with bidomain model predictions of polarization patterns in anisotropic muscle tissue—in particular, tissue with different anisotropy ratios in the intra‐ and extracellular domains [14]. The orientation of the virtual electrodes was the same for serosal and submuscular pacing and was consistent with polarization of the circular muscle layer and not the more superficial longitudinal muscle layer. This further supports the idea that our Vm signals were produced primarily by circular muscle tissue.

Our method could not separate circular muscle polarization from polarization of the ICC‐MY network. However, immunohistology imaging suggests that the ICC‐MY network is at least approximately isotropic [27]. This is supported by fractal analysis of confocal images of the ICC‐MY network, which shows similar succolarity values in three orthogonal directions [28]. Succolarity estimates the potential for transport in a particular direction across an image of a medium. Assuming the ICC‐MY layer is isotropic, ICC‐MY polarization patterns would be expected to be circularly symmetric about the electrode and to lack the side lobes present in the circular muscle [29].

### 4.3 Slow Wave Initiation

Smooth muscle alone does not actively propagate SWs [30]. Rather, smooth muscle cells are electrically connected to the ICC network, probably through gap junctions [31], from which they receive electrical activation [32]. However, in this study, pacing‐elicited SWs did not appear to originate in the ICC‐MY network. If this were the case, SWs from the presumably‐isotropic ICC‐MY network would be expected to arise from circular foci centered on the pacing electrode. Instead, pacing‐elicited slow waves emanated from virtual electrodes lobes that were typically ~0.5–1.0 cm from the pacing electrode. Initiation sites were depolarized virtual cathodes, regardless of the electrical polarity of the pacing electrode. This suggests that pacing‐induced wave initiation was driven by electrical stimulation of the smooth muscle rather than direct stimulation of the ICC network. Our observations are consistent with previous high‐resolution electrical mapping studies, in which the elicited foci were near, but not directly under the pacing sites [7, 8, 33]. Those studies used bipolar pacing, which produce more complex virtual electrode patterns than those produced by the unipolar pacing in the present study [14, 15].

A possible explanation for the wave initiation patterns we observed is that the pacing pulses do not directly activate ICC‐MY tissue under the electrode; rather, they inhibit activation: This might be because both inhibitory and excitatory enteric neurons are present in the gastrointestinal wall and provide input to the ICC network [34]. Electrical stimulation is not specific to any cell population and can activate inhibitory neurons along with other electrically active cells. Pacing has previously been reported to promote inhibitory action in the stomach, probably through vagal and nitrergic pathways [35]. Because the ICC network and network of enteric neurons are both at least approximately isotropic [27, 28, 36], pacing pulses are expected to affect these cells symmetrically about the pacing electrode. On the other hand, our imaging shows that pacing induced asymmetric virtual electrodes in smooth muscle. Although this tissue cannot conduct waves, depolarizing current could flow retrogradely from the depolarized smooth muscle within virtual cathodes into ICC cells that are coupled to them. If the virtual electrode is large enough that these ICC cells are outside the inhibited region close to the pacing electrode, then a wave in the ICC network could initiate and propagate, activating downstream smooth muscle tissue.

The scenario above could underlie the type 0 pacing successes we observed (e.g., Figure 4 and Video S1). In type 1 successes and type 1 failures, activation spread for only a short distance and then subsided. In the above context, this could reflect (1) current spreading for a small distance from the virtual cathodes to neighboring smooth muscle cells combined with (2) failure of depolarizing current from the virtual cathodes into the ICC network to establish a propagating wave. In type 2 failures, activation spread farther—on a centimeter scale—before subsiding. It is possible that such failures reflect successful wave initiation in the ICC network, but with later downstream propagation failure. The mechanism for the secondary focus in type 1 successes is unclear. It could be mechanically mediated, for example, through peg‐and‐socket junctions [37] or neurologically mediated through 2nd messengers [38, 39]. Although not accounting for the majority of successes, type 1 successes were observed in 3 of 4 animals. Their prevalence might be underestimated as two such events were denoted type 2 failures due to later downstream propagation failure.

Refractoriness, a native property of excitable tissue [7, 40], may have been involved in some of the pacing failures we observed. With the fixed 15 s pacing interval, 2:1 pacing capture was observed in some mapping episodes, likely because the 15 s interval was too short for tissue to fully recover between pulses. Alighaleh et al. observed a similar phenomenon [7]. But we also observed both type 0 and type 1 failures occurring immediately before the arrival of an approaching spontaneous wave or after quiescent periods much longer than the native slow wave period that should have been long enough for tissue recovery. It is also worth noting that all sites that suffered pacing failure were activated at other times by either paced or spontaneous slow waves and were therefore capable of supporting slow wave propagation. It is also possible that anesthesia played a role in pacing failure. We previously found that pacing success is more likely during propofol anesthesia than during the isoflurane anesthesia that was used in this study. This could be attributed to the different effects of propofol versus isoflurane on gastric tissue or to higher pacing energy requirements in the presence of dysrhythmic SW patterns that are promoted by isoflurane [41].

Although gastric pacing has promise as a tool for managing motility disorders, it has not yet had significant clinical impact because of its inconsistent efficacy and high energy requirements. Optogenetics is an alternative technology that can be used to manipulate electrically active tissue [42]. Cells that express light‐gated ion channels in their cell membrane can be electrically activated by light pulses. One recent study expressed the protein Channelrhodopsin2 in gastric smooth muscle cells in mice. The authors were able to optically induce smooth muscle contractions that were stronger than contractions elicited by electrical stimulation [43]. A future optical pacing strategy that expresses optogenetic actuator proteins only in targeted cell populations might bypass inhibitory action caused by nonspecific electrical stimulation and enable lower energy and more effective tissue control.

## Author Contributions

H.Z. and J.M.R. conceived and designed research; All authors performed experiments; H.Z., J.M.R., N.D.N., and L.K.C. analyzed data and interpreted results of experiments; H.Z. and J.M.R. prepared figures; H.Z. and J.M.R. drafted manuscript; H.Z., J.M.R., N.D.N., H.N.P., and L.K.C. edited and revised manuscript; All authors approved final version of manuscript.

## Funding

This work was supported by National Institutes of Health (R01EB029428, R21HL140998, and T32EB023872); Royal Society Te Apārangi (20‐UOA‐013‐ILF and 21‐UOA‐180).

## Conflicts of Interest

L.K.C. has intellectual property in the field of gastrointestinal electrophysiology mapping and is a director of FlexiMap Ltd. No financial support was provided by FlexiMap Ltd. for this study. The other authors declare no conflicts of interest.

## Supporting information


**Figure S1:** (A) Plastic frame for preventing tissue deformation during tissue harvesting and fixation. (B) Formalin‐fixed tissue with the fiducial and pacing site markers replaced with transmural Teflon tubes (30AWG, Zeus Industrial Products; black for fiducial markers and yellow for pacing site markers). The pacing sites have been stained with trypan blue to identify muscle fiber orientation.
**Figure S2:** Optical mapping of a type 0 success elicited by an 8 mA, cathodal stimulus at site P. (A) At the onset of pacing, a depolarized virtual cathode is evident, forming two activation foci (red arrows) at the ends of the dogbone‐shaped virtual electrode. (B) Activation at the proximal (bottom‐right) focus subsided, but a SW propagated away from the other focus both distally and proximally on the greater curvature side of the electrode. (C) the elicited SW wavefront continued to propagate and the proximal wave eventually activated the previously depolarized, but failed focus. (D) the elicited SW successfully propagated throughout the entire mapping region. White arrows indicate wavefront propagation direction. (E) The virtual electrode pattern elicited during a 5‐s pacing interval recording with the same pacing location, polarity and strength. The central virtual cathode in E co‐located with the activation foci in A. (F) Optical Vm signals acquired from sites 1–4 in A. Site 1 was at the distal end of the virtual cathode; depolarization occurred immediately upon stimulation and was followed by a full‐blown SW. Site 2 was in one of the virtual anodes; transient hyperpolarization is evident during stimulation; the later depolarization was the result of SW propagation. Site 3 was in the proximal end of the virtual cathode, where the depolarization subsided quickly after the stimulus and the later depolarization was the result of SW propagation. Site 4 was in an area remote from the pacing electrode where the activation was the result of SW propagation.
**Figure S3:** Optical mapping of type 0 success elicited by an 8 mA, cathodal *submuscular* stimulus across the stomach wall from site P. (A) The depolarized virtual cathode (red arrow) was not obscured by the submuscular pacing electrode. (B) Similar to the type 0 success shown in Figure S2, after the stimulus, the activation wavefront at one end of the virtual cathode propagated (white arrows), while depolarization at the other end of virtual cathode subsided. (C and D) The activation continued to propagate to its surroundings in both the proximal and distal directions. White arrows show the wavefront propagation direction. (E) The virtual electrode pattern elicited during a 5‐s pacing interval recording with the same pacing location, polarity and strength. The virtual cathode in E co‐located with the depolarized region in A. (F) Optical Vm signals acquired from sites 1–4 in A. Site 1 was in the virtual cathode. Depolarization was immediate upon stimulation and continued to form a SW. Site 2 was in a virtual anode. Hyperpolarization was evident, but did not affect later depolarization when the wavefront arrived. Sites 3 and 4 were in distal and proximal remote areas and activated by SW propagation.
**Figure S4:** Optical mapping of a type 1 failure of a 4 mA, anodal stimulus. (A and B) Depolarization was evident in virtual cathodes during the stimulus (red arrows). At the same time, a spontaneous retrogradely propagating SW was approaching the pacing site (white arrows). (C) After the pacing stimulus, the stimulus‐induced polarizations quickly subsided. (D) The spontaneous SW passed through the pacing site uninterrupted. (E) The virtual electrode pattern elicited during a 5‐s pacing interval recording with the same pacing location, polarity and strength. The virtual cathodes in E co‐located with the depolarized regions in A. (F) Optical Vm signals acquired from sites 1–4 in A. Propagation of the spontaneous SW is visible in sites 1–4. Sites 2 and 3 are in the virtual anode and cathode, respectively. Transient hyperpolarization and depolarization resulting from stimulus are evident.

**Table S1:** Recordings acquired at each pacing site.

**Table S2:** Number of pacing responses by type and pacing polarity.

**Video S1:** A typical type 0 success. The pacing pulse was anodal. The membrane potential (Vm) was normalized and color coded. The green/yellow dot indicates the location of the pacing electrode (dot turns yellow when pacing pulse is on). The pulse formed two depolarized virtual cathode side lobes about 0.5–1.0 cm away from the pacing electrode that acted as activation foci. SWs spread from these sites.

**Video S2:** A typical type 1 success. The pacing pulse was anodal. The membrane potential (Vm) was normalized and color coded. The green/yellow dot indicates the location of the pacing electrode (dot turns yellow when pacing pulse is on). The pulse formed two depolarized virtual cathode side lobes; one of these lobes initiated a short‐lived SW that propagated a short distance then subsided. About 1–2 s later, a secondary focus arose from the terminal site of the initial short‐lived SW. This SW eventually activated the entire mapping region.

**Video S3:** A typical type 0 failure. The pacing pulse was cathodal. The membrane potential (Vm) was normalized and color coded. The green/yellow dot indicates the location of the pacing electrode (dot turns yellow when pacing pulse is on). A transient depolarization was evident in the dogbone‐shaped virtual cathode, but depolarization quickly subsided without generating a propagating SW.

**Video S4:** A typical type 1 failure. The pacing pulse was cathodal. The membrane potential (Vm) was normalized and color coded. The green/yellow dot indicates the location of the pacing electrode (dot turns yellow when pacing pulse is on). Depolarization at the ends of the dogbone‐shaped virtual cathode are evident. Polarization at one end quickly subsided. At the other end, a transient SW was elicited and propagated for a short distance, primarily along the circular muscular fiber direction, then subsided.

**Video S5:** A typical type 2 failure. The pacing pulse was anodal. The membrane potential (Vm) was normalized and color coded. The green/yellow dot indicates the location of the pacing electrode (dot turns yellow when pacing pulse is on). The two virtual cathodes depolarized and formed foci that merged and propagated to the right (proximally), eventually activating all of the mapping region on that side of the pacing site. However, propagation failed in the distal direction.

## Data Availability

The data that support the findings of this study are available from the corresponding author upon reasonable request.
