# Supplementary material for: Optical Mapping of Pacing‐Elicited Slow Waves in the Swine Stomach: Role of Virtual Electrodes
Source: Neurogastroenterol Motil. 2026 May 5;38:e70340. doi: 10.1111/nmo.70340 (PMC13145316; doi:10.1111/nmo.70340)
Supplement: Supplementary file 1 — Figure S1: (A) Plastic frame for preventing tissue deformation during tissue harvesting and fixation. (B) Formalin‐fixed tissue with the fiducial and pacing site markers replaced with transmural Teflon tubes (30AWG, Zeus Industrial Products; black for fiducial markers and yellow for pacing site markers). The pacing sites have been stained with trypan blue to identify muscle fiber orientation. Figure S2: Optical mapping of a type 0 success elicited by an 8 mA, cathodal stimulus at site P. (A) At the onset of pacing, a depolarized virtual cathode is evident, forming two activation foci (red arrows) at the ends of the dogbone‐shaped virtual electrode. (B) Activation at the proximal (bottom‐right) focus subsided, but a SW propagated away from the other focus both distally and proximally on the greater curvature side of the electrode. (C) the elicited SW wavefront continued to propagate and the proximal wave eventually activated the previously depolarized, but failed focus. (D) the elicited SW successfully propagated throughout the entire mapping region. White arrows indicate wavefront propagation direction. (E) The virtual electrode pattern elicited during a 5‐s pacing interval recording with the same pacing location, polarity and strength. The central virtual cathode in E co‐located with the activation foci in A. (F) Optical Vm signals acquired from sites 1–4 in A. Site 1 was at the distal end of the virtual cathode; depolarization occurred immediately upon stimulation and was followed by a full‐blown SW. Site 2 was in one of the virtual anodes; transient hyperpolarization is evident during stimulation; the later depolarization was the result of SW propagation. Site 3 was in the proximal end of the virtual cathode, where the depolarization subsided quickly after the stimulus and the later depolarization was the result of SW propagation. Site 4 was in an area remote from the pacing electrode where the activation was the result of SW propagation. Figure S3: Optical mappi [file NMO-38-e70340-s003.docx]

# Supporting Information

# Optical Mapping of Pacing-Elicited Slow Waves in the Swine Stomach: Role of Virtual Electrodes

Running Head: Membrane Polarization during Pacing

# Hanyu Zhang^1^, Haley N. Patton^1,4^, Nipuni D. Nagahawatte^2^, Bijay Guragain^1^, Leo K. Cheng^2^, Gregory P. Walcott^3^, Jack M. Rogers^1#^

Affiliations:

^1^ Department of Biomedical Engineering, School of Medicine and School of Engineering, University of Alabama at Birmingham, Birmingham, Alabama, USA.

^2^ Auckland Bioengineering Institute, University of Auckland, New Zealand

^3^ Department of Medicine/Cardiovascular Diseases, University of Alabama at Birmingham, Birmingham, Alabama, USA.

^4^ Present affiliation: Johnson&Johnson MedTech, Biosense Webster, Irvine, California, USA.

^#^Corresponding Author:

Dr. Jack M. Rogers, PhD

Department of Biomedical Engineering

School of Engineering

University of Alabama at Birmingham

E-mail: jrogers@uab.edu

Supporting Figures


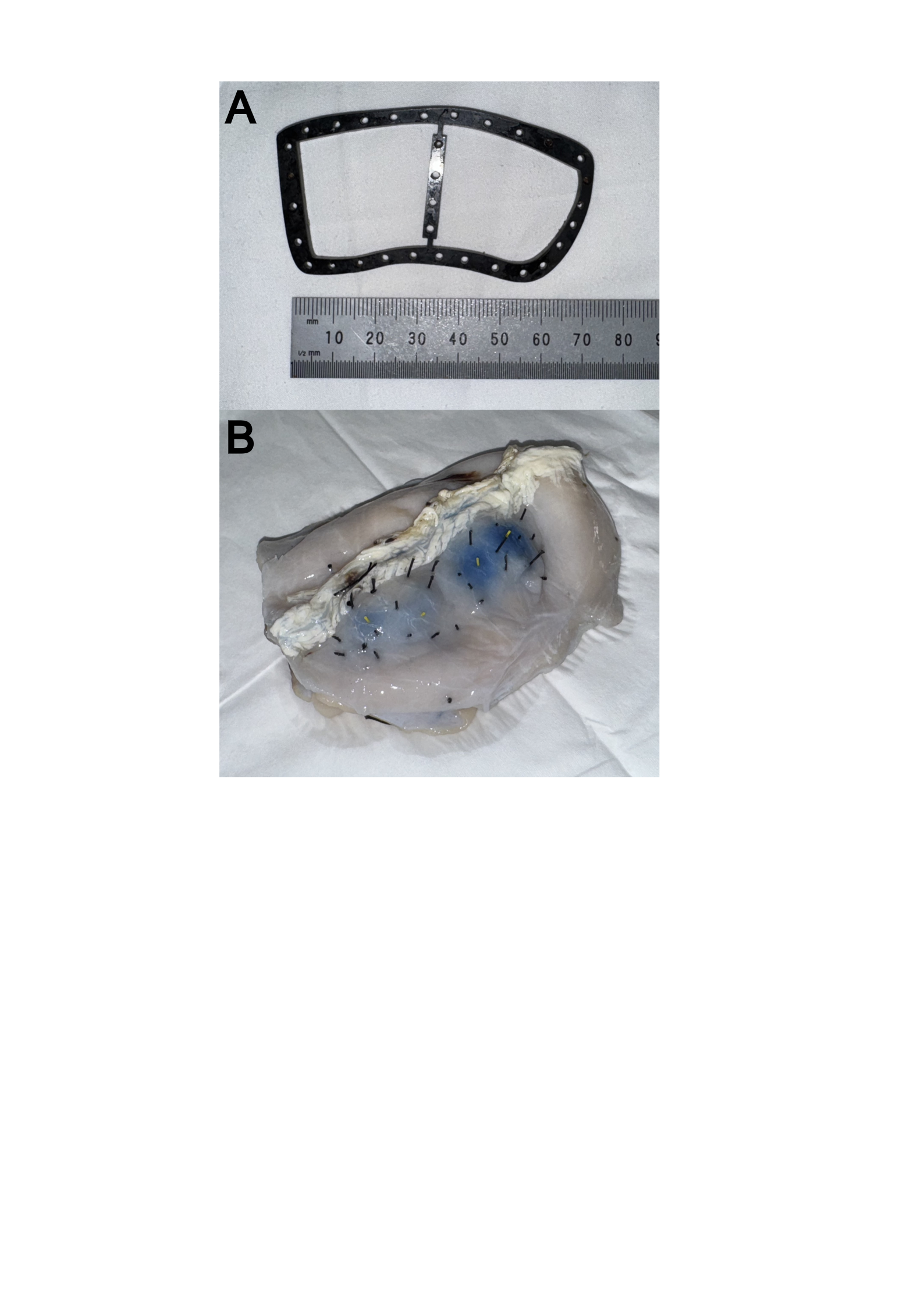


Supporting Figure S1. A: Plastic frame for preventing tissue deformation during tissue harvesting and fixation. B: Formalin-fixed tissue with the fiducial and pacing site markers replaced with transmural Teflon tubes (30AWG, Zeus Industrial Products; black for fiducial markers and yellow for pacing site markers). The pacing sites have been stained with trypan blue to identify muscle fiber orientation.


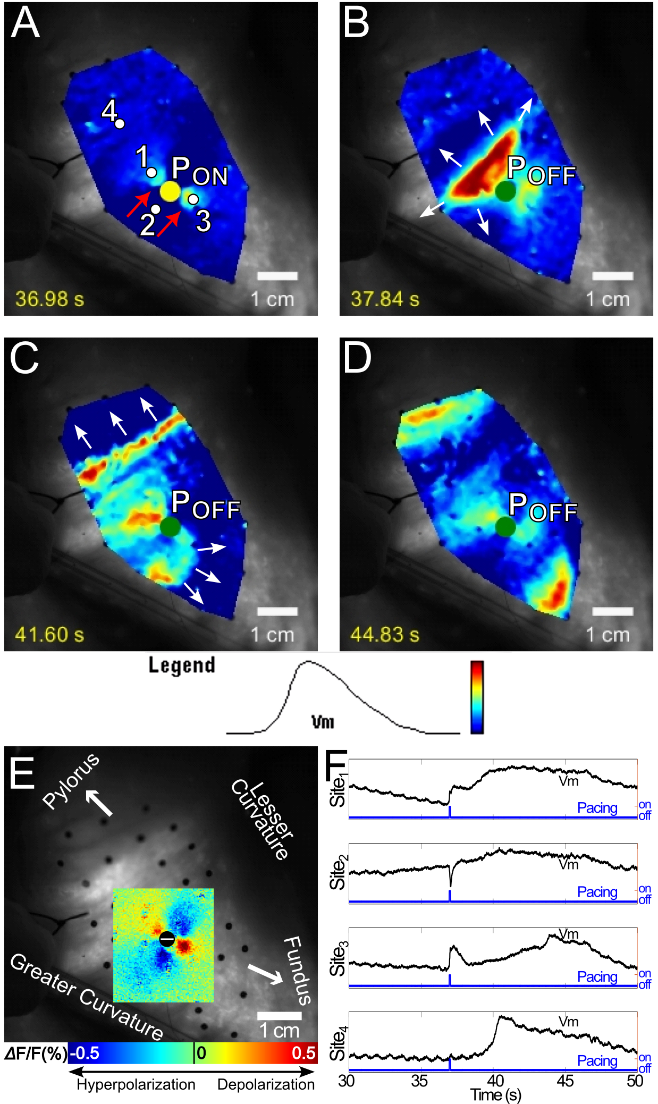


Supporting Figure S2. Optical mapping of a type 0 success elicited by an 8 mA, cathodal stimulus at site P. A: At the onset of pacing, a depolarized virtual cathode is evident, forming two activation foci (red arrows) at the ends of the dogbone-shaped virtual electrode. B: Activation at the proximal (bottom-right) focus subsided, but a SW propagated away from the other focus both distally and proximally on the greater curvature side of the electrode. C: the elicited SW wavefront continued to propagate and the proximal wave eventually activated the previously depolarized, but failed focus. D: the elicited SW successfully propagated throughout the entire mapping region. White arrows indicate wavefront propagation direction. E: The virtual electrode pattern elicited during a 5-sec pacing interval recording with the same pacing location, polarity and strength. The central virtual cathode in E co-located with the activation foci in A. F: Optical Vm signals acquired from sites 1-4 in A. Site 1 was at the distal end of the virtual cathode; depolarization occurred immediately upon stimulation and was followed by a full-blown SW. Site 2 was in one of the virtual anodes; transient hyperpolarization is evident during stimulation; the later depolarization was the result of SW propagation. Site 3 was in the proximal end of the virtual cathode, where the depolarization subsided quickly after the stimulus and the later depolarization was the result of SW propagation. Site 4 was in an area remote from the pacing electrode where the activation was the result of SW propagation.


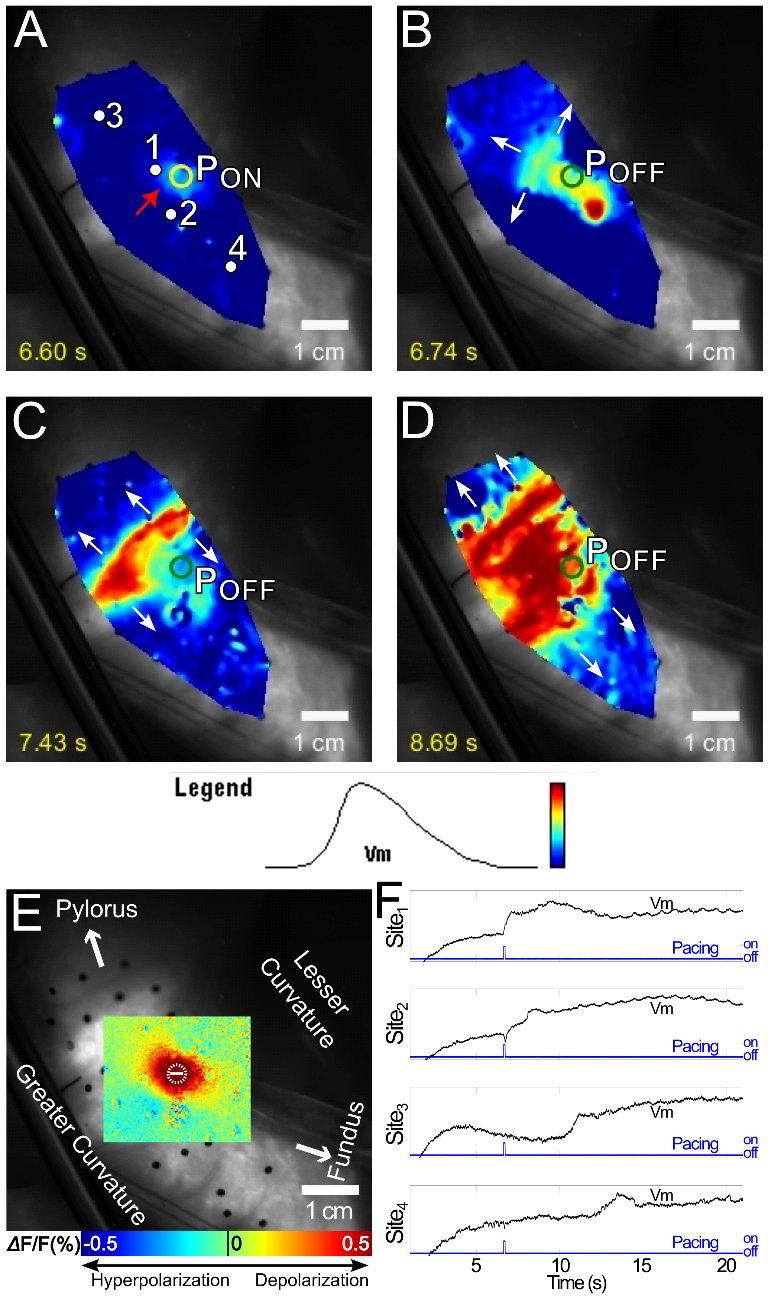


Supporting Figure S3. Optical mapping of type 0 success elicited by an 8 mA, cathodal *submuscular* stimulus across the stomach wall from site P. A: The depolarized virtual cathode (red arrow) was not obscured by the submuscular pacing electrode. B: Similar to the type 0 success shown in Supplemental Figure S2, after the stimulus, the activation wavefront at one end of the virtual cathode propagated (white arrows), while depolarization at the other end of virtual cathode subsided. C and D: the activation continued to propagate to its surroundings in both the proximal and distal directions. White arrows show the wavefront propagation direction. E: The virtual electrode pattern elicited during a 5-sec pacing interval recording with the same pacing location, polarity and strength. The virtual cathode in E co-located with the depolarized region in A. F: Optical Vm signals acquired from sites 1-4 in A. Site 1 was in the virtual cathode. Depolarization was immediate upon stimulation and continued to form a SW. Site 2 was in a virtual anode. Hyperpolarization was evident, but did not affect later depolarization when the wavefront arrived. Sites 3 and 4 were in distal and proximal remote areas and activated by SW propagation.


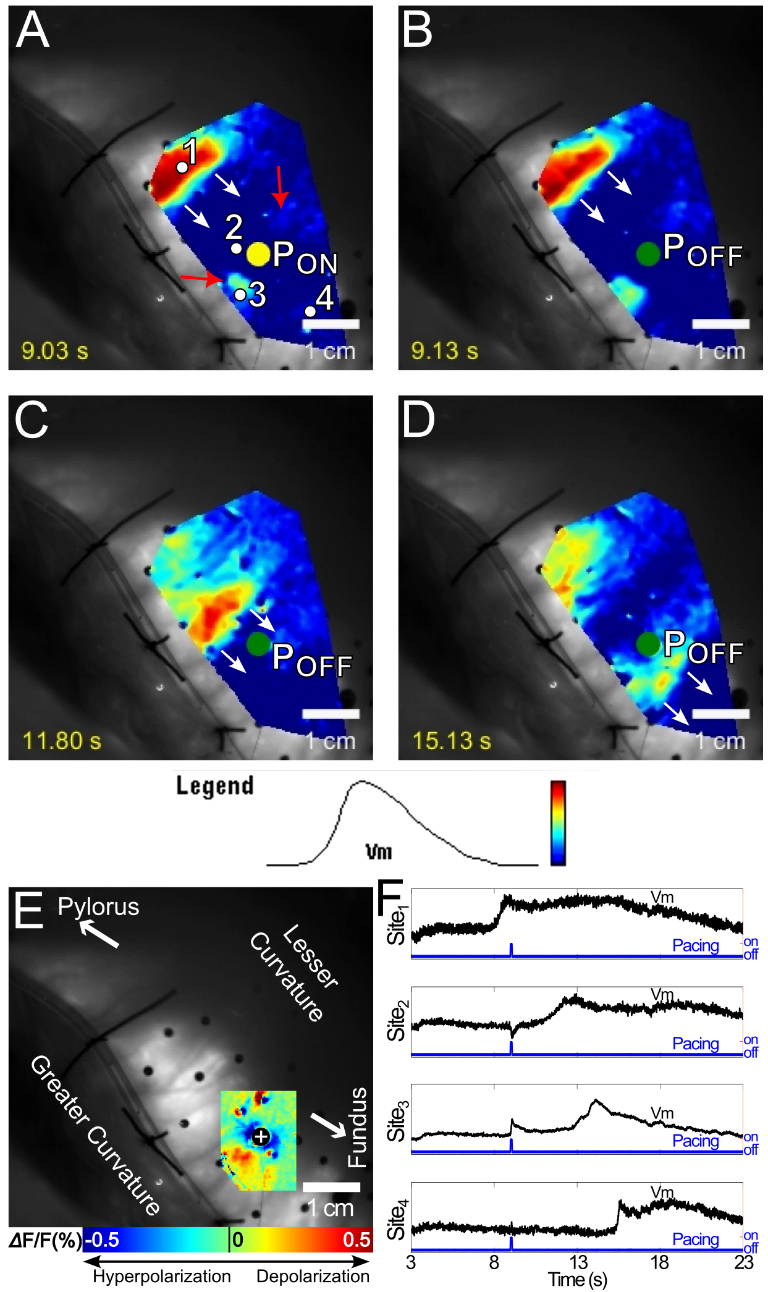


Supporting Figure S4. Optical mapping of a type 1 failure of a 4 mA, anodal stimulus. A and B: depolarization was evident in virtual cathodes during the stimulus (red arrows). At the same time, a spontaneous retrogradely propagating SW was approaching the pacing site (white arrows). C: after the pacing stimulus, the stimulus-induced polarizations quickly subsided. D: the spontaneous SW passed through the pacing site uninterrupted. E: The virtual electrode pattern elicited during a 5-sec pacing interval recording with the same pacing location, polarity and strength. The virtual cathodes in E co-located with the depolarized regions in A. F: optical Vm signals acquired from sites 1-4 in A. Propagation of the spontaneous SW is visible in sites 1-4. Sites 2 and 3 are in the virtual anode and cathode, respectively. Transient hyperpolarization and depolarization resulting from stimulus are evident.
