# Supplementary material for: Optical Mapping of Pacing‐Elicited Slow Waves in the Swine Stomach: Role of Virtual Electrodes
Source: Neurogastroenterol Motil. 2026 May 5;38:e70340. doi: 10.1111/nmo.70340 (PMC13145316; doi:10.1111/nmo.70340)
Supplement: Supplementary file 2 — Table S1: Recordings acquired at each pacing site. [file NMO-38-e70340-s001.docx]

| **Table S1. Recordings Acquired at Each Pacing Site** | | | |
| --- | --- | --- | --- |
| **Order** | **Pulse Amplitude (mA)** | **Cycle Length (s)** | **Polarity** |
| 1 | No Pacing | | |
| 2 | 4 | 15 | Anodal |
| 3 | 4 | 15 | Cathodal |
| 4 | 4 | 5 | Anodal |
| 5 | 4 | 5 | Cathodal |
| 6 | 8 | 15 | Anodal |
| 7 | 8 | 15 | Cathodal |
| 8 | 8 | 5 | Anodal |
| 9 | 8 | 5 | Cathodal |
| 10 | No Pacing | | |
