# Supplementary material for: Optical Mapping of Pacing‐Elicited Slow Waves in the Swine Stomach: Role of Virtual Electrodes
Source: Neurogastroenterol Motil. 2026 May 5;38:e70340. doi: 10.1111/nmo.70340 (PMC13145316; doi:10.1111/nmo.70340)
Supplement: Supplementary file 3 — Table S2: Number of pacing responses by type and pacing polarity. [file NMO-38-e70340-s006.docx]

| **Table S2. Number of Pacing Responses by Type and Pacing Polarity** | | | | | | | | | | |
| --- | --- | --- | --- | --- | --- | --- | --- | --- | --- | --- |
|  | **Type 0 Success** | | **Type 1 Success** | | **Type 0 Failure** | | **Type 1 Failure** | | **Type 2 Failure** | |
| **Pig** | **Anodal** | **Cathodal** | **Anodal** | **Cathodal** | **Anodal** | **Cathodal** | **Anodal** | **Cathodal** | **Anodal** | **Cathodal** |
| 1 | 6 | 3 | 0 | 0 | 7 | 16 | 6 | 0 | 3 | 0 |
| 2 | 0 | 0 | 0 | 1 | 2 | 21 | 26 | 6 | 3 | 0 |
| 3 | 11 | 5 | 0 | 1 | 4 | 2 | 4 | 9 | 4 | 4 |
| 4 | 3 | 0 | 2 | 0 | 3 | 12 | 8 | 1 | 2 | 0 |
| All | 20 (11%) | 8 (5%) | 2 (1%) | 2 (1%) | 16 (9%) | 51 (29%) | 44 (25%) | 16 (9%) | 12 (7%) | 4 (2%) |
