# Supplementary material for: Optical Mapping of Pacing‐Elicited Slow Waves in the Swine Stomach: Role of Virtual Electrodes
Source: Neurogastroenterol Motil. 2026 May 5;38:e70340. doi: 10.1111/nmo.70340 (PMC13145316; doi:10.1111/nmo.70340)
Supplement: Supplementary file 4 — Video S1: A typical type 0 success. The pacing pulse was anodal. The membrane potential (Vm) was normalized and color coded. The green/yellow dot indicates the location of the pacing electrode (dot turns yellow when pacing pulse is on). The pulse formed two depolarized virtual cathode side lobes about 0.5–1.0 cm away from the pacing electrode that acted as activation foci. SWs spread from these sites. [file NMO-38-e70340-s005.zip › Supporting Video S1.docx]

Supporting Video S1: A typical type 0 success. The pacing pulse was anodal. The membrane potential (Vm) was normalized and color coded. The green/yellow dot indicates the location of the pacing electrode (dot turns yellow when pacing pulse is on). The pulse formed two depolarized virtual cathode side lobes about 0.5-1.0 cm away from the pacing electrode that acted as activation foci. SWs spread from these sites.
