# Supplementary material for: Optical Mapping of Pacing‐Elicited Slow Waves in the Swine Stomach: Role of Virtual Electrodes
Source: Neurogastroenterol Motil. 2026 May 5;38:e70340. doi: 10.1111/nmo.70340 (PMC13145316; doi:10.1111/nmo.70340)
Supplement: Supplementary file 5 — Video S2: A typical type 1 success. The pacing pulse was anodal. The membrane potential (Vm) was normalized and color coded. The green/yellow dot indicates the location of the pacing electrode (dot turns yellow when pacing pulse is on). The pulse formed two depolarized virtual cathode side lobes; one of these lobes initiated a short‐lived SW that propagated a short distance then subsided. About 1–2 s later, a secondary focus arose from the terminal site of the initial short‐lived SW. This SW eventually activated the entire mapping region. [file NMO-38-e70340-s002.zip › Supporting Video S2.docx]

Supporting Video S2: A typical type 1 success. The pacing pulse was anodal. The membrane potential (Vm) was normalized and color coded. The green/yellow dot indicates the location of the pacing electrode (dot turns yellow when pacing pulse is on). The pulse formed two depolarized virtual cathode side lobes; one of these lobes initiated a short-lived SW that propagated a short distance then subsided. About 1-2 seconds later, a secondary focus arose from the terminal site of the initial short-lived SW. This SW eventually activated the entire mapping region.
