# Supplementary material for: Optical Mapping of Pacing‐Elicited Slow Waves in the Swine Stomach: Role of Virtual Electrodes
Source: Neurogastroenterol Motil. 2026 May 5;38:e70340. doi: 10.1111/nmo.70340 (PMC13145316; doi:10.1111/nmo.70340)
Supplement: Supplementary file 6 — Video S3: A typical type 0 failure. The pacing pulse was cathodal. The membrane potential (Vm) was normalized and color coded. The green/yellow dot indicates the location of the pacing electrode (dot turns yellow when pacing pulse is on). A transient depolarization was evident in the dogbone‐shaped virtual cathode, but depolarization quickly subsided without generating a propagating SW. [file NMO-38-e70340-s007.zip › Supporting Video S3.docx]

Supporting Video S3: A typical type 0 failure. The pacing pulse was cathodal. The membrane potential (Vm) was normalized and color coded. The green/yellow dot indicates the location of the pacing electrode (dot turns yellow when pacing pulse is on). A transient depolarization was evident in the dogbone-shaped virtual cathode, but depolarization quickly subsided without generating a propagating SW.
