# Supplementary material for: Optical Mapping of Pacing‐Elicited Slow Waves in the Swine Stomach: Role of Virtual Electrodes
Source: Neurogastroenterol Motil. 2026 May 5;38:e70340. doi: 10.1111/nmo.70340 (PMC13145316; doi:10.1111/nmo.70340)
Supplement: Supplementary file 7 — Video S4: A typical type 1 failure. The pacing pulse was cathodal. The membrane potential (Vm) was normalized and color coded. The green/yellow dot indicates the location of the pacing electrode (dot turns yellow when pacing pulse is on). Depolarization at the ends of the dogbone‐shaped virtual cathode are evident. Polarization at one end quickly subsided. At the other end, a transient SW was elicited and propagated for a short distance, primarily along the circular muscular fiber direction, then subsided. [file NMO-38-e70340-s008.zip › Supporting Video S4.docx]

Supporting Video S4: A typical type 1 failure. The pacing pulse was cathodal. The membrane potential (Vm) was normalized and color coded. The green/yellow dot indicates the location of the pacing electrode (dot turns yellow when pacing pulse is on). Depolarization at the ends of the dogbone-shaped virtual cathode are evident. Polarization at one end quickly subsided. At the other end, a transient SW was elicited and propagated for a short distance, primarily along the circular muscular fiber direction, then subsided.
