# Supplementary material for: Optical Mapping of Pacing‐Elicited Slow Waves in the Swine Stomach: Role of Virtual Electrodes
Source: Neurogastroenterol Motil. 2026 May 5;38:e70340. doi: 10.1111/nmo.70340 (PMC13145316; doi:10.1111/nmo.70340)
Supplement: Supplementary file 8 — Video S5: A typical type 2 failure. The pacing pulse was anodal. The membrane potential (Vm) was normalized and color coded. The green/yellow dot indicates the location of the pacing electrode (dot turns yellow when pacing pulse is on). The two virtual cathodes depolarized and formed foci that merged and propagated to the right (proximally), eventually activating all of the mapping region on that side of the pacing site. However, propagation failed in the distal direction. [file NMO-38-e70340-s004.zip › Supporting Video S5.docx]

Supporting Video S5: A typical type 2 failure. The pacing pulse was anodal. The membrane potential (Vm) was normalized and color coded. The green/yellow dot indicates the location of the pacing electrode (dot turns yellow when pacing pulse is on). The two virtual cathodes depolarized and formed foci that merged and propagated to the right (proximally), eventually activating all of the mapping region on that side of the pacing site. However, propagation failed in the distal direction.
